# Correction: Rodríguez-Medellín, R., et al. Mexican Validation of the Engagement and Disaffection in Physical Education Scale. *Int. J. Environ. Res. Public Health* 2020, *17*, 1821

**DOI:** 10.3390/ijerph18020782

**Published:** 2021-01-18

**Authors:** René Rodríguez-Medellín, Jorge Zamarripa, María Marentes-Castillo, Fernando Otero-Saborido, Raúl Baños, Raquel Morquecho-Sánchez

**Affiliations:** 1 Faculty of Sports Organization, Autonomous University of Nuevo Leon, Cd. Universitaria, s/n, San Nicolás de los Garza, Nuevo Leon 66451, Mexico; rene.rodriguezmd@uanl.edu.mx (R.R.-M.); salette.marentescs@uanl.edu.mx (M.M.-C.); raquel.morquechosn@uanl.edu.mx (R.M.-S.); 2 Faculty of Sports Sciences, Pablo de Olavide University, Ctra. de Utrera, 1, 41013 Sevilla, Spain; fmotero@upo.es; 3 Campus Ensenada, Faculty of Sports, Autonomous University of Baja California, Paseo de la Playa, Carlos Pacheco 7, Ensenada 22890, Mexico; raulfb89@gmail.com

The authors wish to add the following correction to their paper published in International Journal of Environmental Research and Public Health [1].

In row 2 of Table 1 (page 5), “Emotional engagement” appears in italics, this should instead be “Behavioral engagement”.

In row 7 of Table 1 (page 5), “Behavioral engagement” appears in italics, this should instead be “Emotional engagement”.

In row 15 of Table 1 (page 5), “Emotional disaffection” appears in italics, this should instead be “Behavioral disaffection”.

In row 21 of Table 1 (page 5), “Behavioral disaffection” appears in italics, this should instead be “Emotional disaffection”.

The authors would like to apologize for any confusion or inconvenience. Corrected Table 1 is presented below.

## Figures and Tables

**Table 1:** The standardized solution of the four sub-scales of the instrument.

Sub-Scales		*M*	*SD*	*Asymmetry*	*Kurtosis*	Factorial Saturations	
						*2 Factors*	*4 Factors*
*Engagement*		4.10	0.72	−1.10	1.49		
	*Behavioral engagement*	4.21	0.85	−1.26	1.33		
1	I pay attention in my physical education class (*Pongo atención en la clase de educación física*)	4.38	1.01	−1.81	2.76	0.64	0.67
2	I study for my physical education class (*Estudio para la clase de educación física*)	3.25	1.51	−0.35	−1.32	0.24	0.26
3	I try to do the most I can in the physical education class (*Trato de hacer lo más que pueda en la clase de educación física*)	4.35	0.98	−1.62	2.12	0.74	0.80
	*Emotional engagement*	3.99	0.82	−0.78	0.49		
4	I enjoy the time I spend in the physical education class (*Disfruto del tiempo que paso en la clase de educación física*)	4.42	1.00	−1.90	3.01	0.77	0.79
5	It is exciting when I make connections between ideas learned in the physical education class (*Es emocionante cuando hago conexiones entre las ideas aprendidas en la clase de educación física*)	4.09	1.14	−1.22	0.72	0.64	0.64
6	The content we see in the physical education class is interesting (*Es interesante el contenido que vemos en la clase de educación física*)	4.12	1.16	−1.28	0.74	0.69	0.69
*Disaffection*		2.21	1.05	0.72	−0.34		
	*Behavioral disaffection*	1.93	1.20	1.09	−0.07		
7	It is difficult to attend the physical education class (*Es difícil asistir a la clase de educación física*)	2.25	1.50	0.72	−1.04	0.61	0.71
8	I only do enough to pass the physical education class (*Sólo hago lo suficiente para pasar en la clase de educación física*)	2.85	1.60	0.09	−1.56	0.44	0.59
9	I do not do much work outside the physical education class (*No hago mucho trabajo fuera de la clase de educación física*)	2.35	1.48	0.60	−1.12	0.66	0.78
	*Emotional disaffection*	1.93	1.20	1.09	−0.07		
10	The classes of the physical education teacher are very boring (*Son muy aburridas las clases del profesor de educación física*)	1.97	1.37	1.10	−0.23	0.86	0.86
11	The physical education class stresses me (*Me estresa la clase de educación física*)	2.00	1.41	1.09	−0.33	0.86	0.86
12	Being in the physical education class is a waste of time (*Es una pérdida de tiempo estar en la clase de educación física*)	1.81	1.34	1.43	0.57	0.90	0.91

*Note*: *M*—Mean; *SD*—Standard Deviation. All saturations were significant, *t* > 1.96, *p* < 0.05.
